# Re‐engagement, quality of life, and burden of treatment in adults on dupilumab for severe atopic dermatitis—A mixed methods study

**DOI:** 10.1002/ski2.372

**Published:** 2024-03-21

**Authors:** Emma Porter, Cathal O’Connor, Michelle Murphy

**Affiliations:** ^1^ Dermatology South Infirmary Victoria University Hospital Cork Ireland; ^2^ Dermatology University College Cork Cork Ireland; ^3^ INFANT Research Centre University College Cork Cork Ireland

## Abstract

**Background:**

Targeted biologic therapies have revolutionised the treatment of severe atopic dermatitis (AD).

**Objectives:**

To assess effects of dupilumab on patient re‐engagement, quality of life (QOL), and burden of treatment (BOT) in severe AD.

**Methods:**

Adults on dupilumab for AD completed questionnaires on QOL, BOT, and provided qualitative reflections, with a subset interviewed to explore experience of leaving and re‐engaging with dermatology. Prior treatments, adverse events, and clinical severity scoring were evaluated. Statements and interviews were qualitatively reviewed.

**Results:**

Of 41 patients; median age was 34 years, 68% were male; and 93% (*n* = 38) had trialled ≥1 immunomodulatory therapies before dupilumab. Median dermatology life quality index was 21 (range 9–30, SD ± 5.1) pre‐dupilumab, and 2 (range 0–11, SD ± 3.4) post‐dupilumab. Median eczema area and severity index was 31.4 (range 10–46.4, SD ± 11.8) pre‐dupilumab, and 6.4 (range 0.4–13.2, SD ± 3.6) on dupilumab. Burden of treatment scores on dupilumab were low (median 0–3/10) across all domains. Themes identified pre‐dupilumab included sleep disturbance, low self‐esteem, social isolation, disempowerment, frustration with ineffective treatments, and high financial costs. Benefits included confidence reacquisition, enhanced sleep, liberation from time‐consuming ‘messy’ topical regimes, improved relationships, and reclaimed autonomy. Side effects included red/itchy eyes (37%, *n* = 13) and facial dermatitis (20%, *n* = 7).

Twelve patients had deeper interviews. Regarding disengagement with dermatology, themes included ineffectiveness and toxicity of older treatments, attendance futility, dermatologist fatigue, and ‘fizzling out’. Regarding re‐engagement with dermatology, themes included social media influence, novelty, exasperation with QOL, and life‐changing improvements seen with dupilumab.

**Conclusions:**

The emergence of novel effective treatments for AD has significant implications for dermatology workforce and financial planning.

## Graphical abstract

Targeted biologic therapies have revolutionised the treatment of severe atopic dermatitis (AD). The aim of this study was to assess effects of dupilumab on patient re‐engagement, quality of life, and burden of treatment in severe AD. Patients described poor quality of life before dupilumab, life‐changing changes with dupilumab, and reasons why they left and re‐engaged with dermatology care.

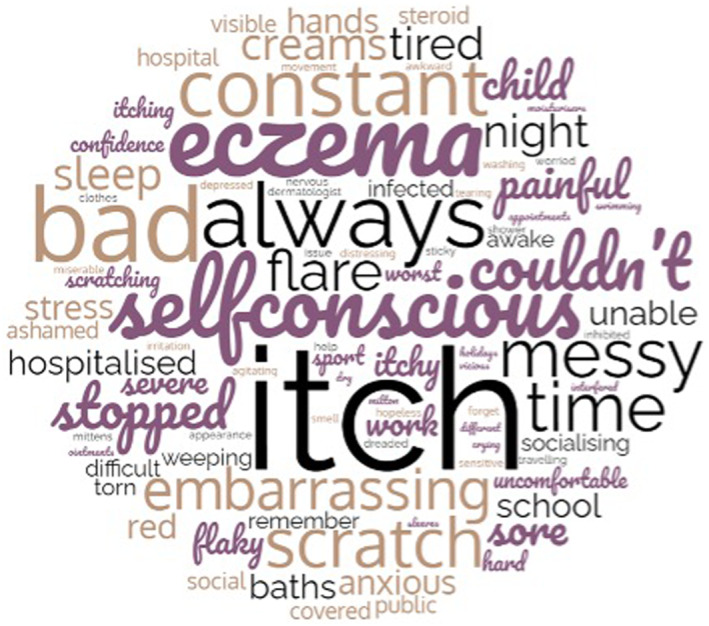

**What is already known about this topic?**

- Atopic dermatitis (AD) significantly disrupts patients' quality of life (QOL), with an associated high burden of treatment (BOT). Until recently, therapeutic options for severe AD were limited, leaving many patients with severe AD exasperated with dermatology care. However, the availability of novel treatments, such as dupilumab, has revolutionised the management of severe AD, leading to an influx of patients with severe AD returning to dermatology services.

**What does this study add?**

- This mixed methods study assessed the effect of dupilumab on QOL and BOT in patients with severe AD, and also qualitatively examined the reasons why patients left and re‐engaged with dermatology care. Patients reported low BOT and transformative effects on QOL after commencing dupilumab, and explained that they left dermatology care because of ineffective treatments and re‐engaged because of novel effective therapies.

**What are the clinical implications of this work?**

- This study adds to the understanding of the lived patient experience of severe AD and the profound biopsychosocial benefit of effective treatment. Patients on dupilumab report low BOT in requirements for topical treatment, monitoring, healthcare utilization, and financial costs. The implications of patients returning to dermatology care for novel therapies should be factored into healthcare workforce and financial planning.

## 1 INTRODUCTION

Atopic dermatitis (AD) has a devastating impact on quality of life (QOL),^1^ and persistent AD affects 7% of adults.^2^ The major disabling and burdensome features of severe AD in adults include itch, sleep disruption, cognitive dysfunction, low mood, work or education impairment, impact on social or sexual relationships, cosmetic appearance, time spent on treatment, and effects on activities of daily living.^3^ Over half of adults with AD are at risk of therapeutic burnout due to excessive burden of treatment (BOT),^4^ due to the need for regular topical regimes, financial implications, frequent hospital or clinic visits, potential side effects and medication monitoring. However, parents of children with severe AD have reported that effective systemic treatment can reduce BOT.^5^ Until recently, options for severe AD beyond topical therapies were limited to phototherapy and conventional immunosuppressive systemic drugs such as methotrexate and ciclosporin.^6^ Dupilumab, a monoclonal antibody blocking interleukin (IL)−4 and IL‐13 via their shared IL‐4α subunit, has demonstrated clinical efficacy and safety in treating moderate‐to‐severe AD.^7^, ^8^, ^9^ Moreover, dupilumab has been shown to improve depression and anxiety related to severe AD,^10^ although the degree of improvement in mental health may not directly correlate with improvement in AD severity.^11^ With the advent of treatments such as dupilumab and janus kinase inhibitors (JAKi), patients with AD who had lost follow up with dermatology are now returning in increasing numbers for consideration of effective treatments.^12^ The aim of this study was to quantitively and qualitatively analyse the impact of dupilumab on QOL and BOT, and to qualitatively assess the recapture of adults with severe AD who had previously been lost to follow up from dermatology because of disillusionment with suboptimal therapies.

## 2 METHODS

Ethical approval was authorised by the Clinical Research Ethics Committee of the Cork Teaching Hospital (reference number ECM 4 (i) 14/02/2023). For the questionnaire component of the study, adults on dupilumab for severe AD in our dermatology outpatient clinic were invited to complete questionnaires during scheduled care. Severe AD was defined as investigator global assessment score of 4,^13^ or eczema area and severity index (EASI) of >21,^14^ prior to initiation of therapy with dupilumab. Questionnaires included a dermatology life quality index (DLQI),^15^ a validated BOT questionnaire,^16^ and a qualitative section asking for free‐text reflections on impact of dupilumab on QOL. BOT questionnaire items relate to treatments, follow‐up, financial and administrative burden and is scored 0–10, with 0 being ‘not a problem’ and 10 ‘a big problem’.^16^ Statements were qualitatively reviewed, identifying common themes. Data regarding prior treatments, adverse events and clinical severity scoring were collected. Semi‐structured interviews were performed by telephone with a subset of adults with severe AD to discuss their pathway to return to dermatology. Patients were included if they had been re‐referred back to dermatology and had started therapy with dupilumab for severe AD. Questionnaires were analysed quantitatively using Microsoft 365 and SPSS, while qualitative statements were analysed using Microsoft 365 and NVIVO.

## 3 RESULTS

**FIGURE 1:**
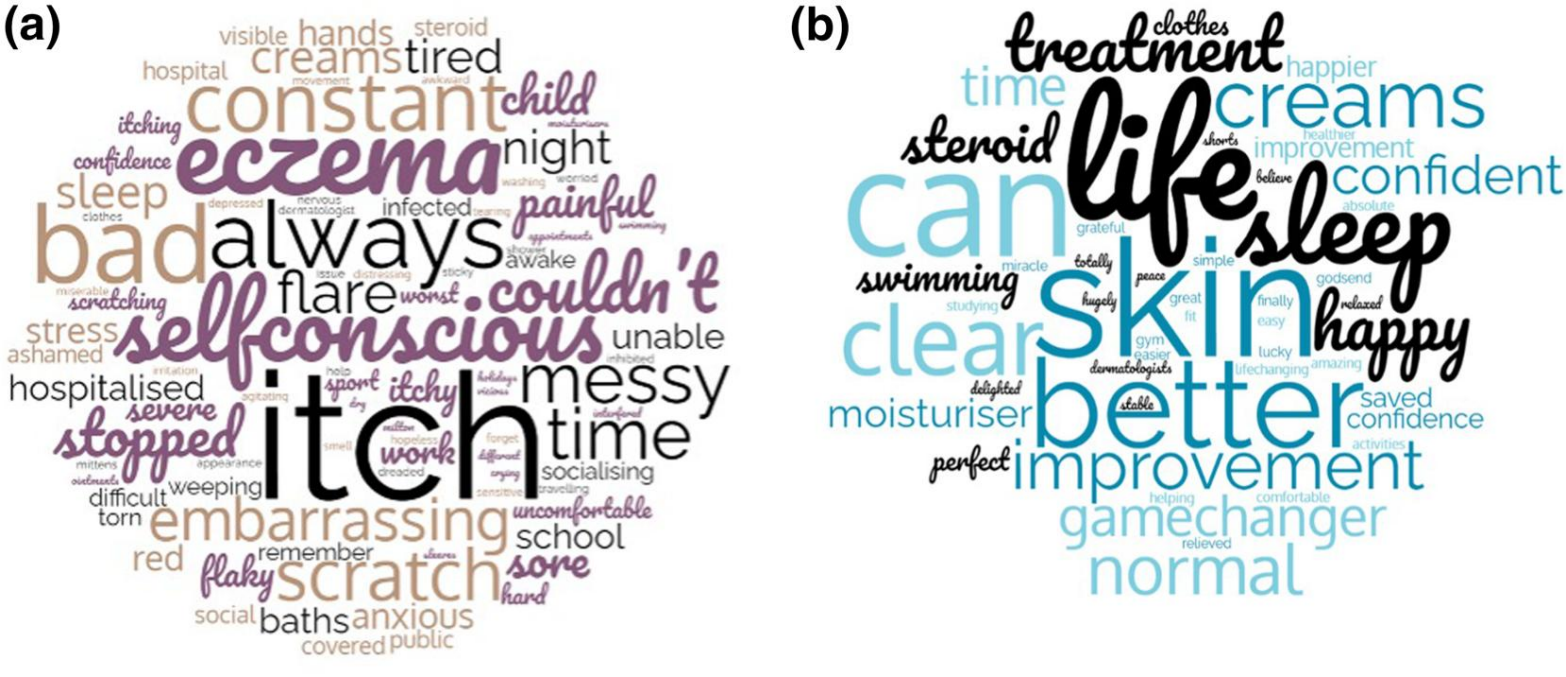
(a) and (b) Word clouds illustrating frequency of words used by patients in questionnaires, reflecting on quality of life before starting dupilumab (left) and while on treatment (right).

Forty‐one patients participated in the questionnaire study. Median age of patients was 34 (range 18–71), and 68% (*n* = 28) were male. Thirty‐eight patients (93%) had therapeutic failure with at least one immunosuppressant medication prior to dupilumab, and eight (20%) had failure with ≥2 agents. Dupilumab was prescribed as per standard licenced dosing (600 mg loading dose, then 300 mg every 2 weeks). The median duration of treatment with dupilumab was 15 months (range 3–61 months). Median DLQI was 21 (range 9–30, SD ± 5.1) pre dupilumab, and 2 (range 0–11, SD ± 3.4) on dupilumab. Median EASI score was 31.4 (range 10–46.4, SD ± 11.8) pre‐dupilumab, and 6.4 (range 0.4–13.2, SD ± 3.6) on dupilumab. Average BOT scores on dupilumab were low (0/10‐3/10) across all domains, with an average global BOT score of 16/150, which is rated as very low.^17^ Themes identified in pre‐dupilumab reflections (Table 1) included sleep disturbance, low self‐esteem, stress, anxiety, social isolation, disempowerment, frustration with ineffective treatments, negative childhood memories, and high financial costs. Patients described dramatic improvements on dupilumab: ‘I spent years like a recluse, I was hopeless—this completely transformed my life’. Themes related to benefits of dupilumab included enhanced sleep, liberation from time‐consuming ‘messy’ topical regimes, freedom of clothing choices, improved relationships, confidence reacquisition, renewed enjoyment of pastimes, and reclaimed autonomy (Table 2). Word clouds representing patients' frequently used words to describe their experience pre and post dupilumab are illustrated in Figure 1. Patients did report disappointment with some side effects, including red/itchy/dry eyes (34%, *n* = 14), facial dermatitis (20%, *n* = 8), and recurrent herpes labialis (2%, *n* = 1). There were no significant differences in mean BOT scores among those who reported adverse ocular or facial effects.

**TABLE 1:** Themes identified in patient reflections of quality of life prior to starting dupilumab.

Theme	Illustrative patient statements
Sleep disturbance	‘My bed used to be covered in blood and flakes of skin from scratching all night.’
	‘At its worst, I was awake all night itching.’
Low self‐esteem	‘I had no confidence, I didn't go out, I was socially awkward especially when my face was bad.’
	‘It was hard going out feeling ashamed of how I looked and wanting to scratch.’
Social isolation	‘I kept away from everyone. Not just because I was embarrassed, but I was so irritable that I wasn't a nice person to be around.’
	‘I felt left out of the normal things my friends would do because I would have to stay home when my eczema was bad.’
Disempowerment	‘Eczema restricted everything. I couldn't really do anything when my skin was bad.’
	‘For a long time I just gave up and stopped coming to appointments, because I felt so hopeless that nothing was going to work for me.’
Frustration with ineffective treatments	‘I spent my 20s covered in sticky creams and having to have nightly baths.’
	‘I went through every single treatment you can think of for eczema and barely anything worked. Some did help, but I got side effects and had to stop.’
Financial impact	‘I was spending hundreds of euros on moisturizers and steroid creams that I hated in the first place.’
	‘I had to take time off work and don't get sick pay, so every time my eczema flared I was losing money.’
Negative childhood memories	‘I was pretty much born with eczema. I have mittens on in all my baby photos to stop me scratching.’
	‘I was hospitalised multiple times as a child. I missed so much of primary school because of my skin.’
	‘Bleach baths as a child were horrible, I still remember the smell and crying with my skin so sore.’
Stress and anxiety	‘It was always in my head, it depressed me.’
	‘I was anxious all the time, miserable altogether.’

**TABLE 2:** Themes identified in patient reflections of quality of life after commencing dupilumab and illustrative patient statements.

Theme	Illustrative patient statements
Enhanced sleep	‘I can sleep through the night and not wake up sore.’
	‘Being able to sleep at night changes everything. I'm like a whole new person!’
Confidence reacquisition	‘I can't even remember what it was like to feel as embarrassed as I did before.’
	‘I used to not go out, now I'm confident again and I'm not worried about looking red. And I don't put my hood up anymore whenever I go out of the house.’
Improved relationships	‘My social life is a million times better.’
	‘I don't keep my wife awake all night with my scratching now, which was really disruptive.’
	‘I can take my kids swimming at the pool and enjoy it instead of stressing about my skin.’
Reclaimed autonomy	‘It's a real eye opener. I thought I would have my entire life ahead of me as a socially awkward person with skin problems, but this has given me a new lease of life.’
	‘I've been given back a normal life.’
Liberation from topical regimes	‘I only need to use creams twice a week. It has saved me so much time day to day.’
	‘I haven't used a steroid ointment since my first injection over a year ago!’
	‘My clothes don't stick to me anymore.’
Freedom of clothing and appearance choices	‘I can wear shorts and t‐shirts again—before I used to keep long sleeves on to cover my skin even in the summer.’
	‘I am so happy to be able to wear makeup again, and fake tan isn't patchy or flaky on me now.’
	‘Before I could never wear anything nice, now I can express my style a bit more.’
Renewed enjoyment of activities	‘It's a massive change mentally. I can do the things in life I love—gym, swimming, outdoor activities.’
	‘I have planned the first family holiday in years that I can look forward to, I'll be able to go to the beach and pool and not have to worry about messy creams.’
	‘I've gone back to the gym and started looking after my health in general better.’

Semi‐structured interviews were conducted by telephone with 12 patients (six men and six women) with severe AD who had started dupilumab (Table 3). The average age was 48 years (range 19–70). Almost all patients had previously undergone phototherapy (*n* = 11), and all had treatment failure with methotrexate (*n* = 12). Three patients had previous skin cancer, and seven had asthma. The average number of previous systemic immunomodulatory therapies was 2 (range 0–4). Semi‐structured questions were divided into two categories of concepts—experiences of losing contact with dermatology and experiences of re‐engagement with dermatology.

**TABLE 3:** Demographic and clinical details of patients interviewed regarding experience of disengagement and re‐engagement with dermatology care.

	Sex	Age	Co‐morbidities	Previous Tx
1	M	70	Cutaneous SCC	UVB, PUVA, MTX
2	M	66	Asthma, HSV keratitis, hypertension	UVB, MTX, AZA, MMF
3	F	54	Asthma	UVB, MTX, CSA, MMF, AZA
4	F	52	Contact dermatitis, asthma	UVB, MTX
5	M	28	Asthma	UVB, MTX, UPA
6	M	29	Asthma	UVB, MTX, UPA
7	M	36	Allergic rhinitis	MTX
8	F	23	Asthma	UVB, MTX, CSA
9	F	19	Nil	UVB, MTX
10	F	61	Latent TB	UVB, MTX, CSA, AZA, MMF, UPA
11	M	72	Asthma, hypertension, cutaneous SCC	UVB, MTX, CSA, MMF, TOF
12	F	63	Melanoma in situ, actinic keratoses	UVB, MTX, UPA,TOF

Abbreviations: AZA, azathioprine; CSA, ciclosporin; HSV, herpes simplex virus; MMF, mycophenolate mofetil; MTX, methotrexate; PUVA, Psoralen + ultraviolet A; SCC, squamous cell carcinoma; TB, tuberculosis; UPA, upadacitinib; UVB, ultraviolet B.

Themes related to leaving dermatology care included.

(i) Ineffectiveness of older treatments

I was told that phototherapy would be great but I spent months and months coming and going doing lights treatments, and I was nearly worse off at the end of the three months than I was before.

Methotrexate made things slightly better in that I didn’t have to use so many creams, but it didn’t really improve things substantially.

(ii) Toxicity of older treatments

The ciclosporin would work for a few weeks but then I would have a flare, and my kidneys nearly gave out.

No matter what I tried to do I felt so sick from the methotrexate, the nausea was terrible.

(iii) Attendance futility

I remember the last clinic I went to, I was sitting for an hour in the waiting room thinking ‘what is the point of keeping coming here’ when all I was told to do was use another cream or try a different bath.

(iv) Dermatologist fatigue

I could tell the dermatologist was sick of looking at me. Back then I thought I was the problem, but now I think they were embarrassed because they couldn’t help me.

I felt like the doctors were just going through the motions, just prescribing the same stuff that had stopped working.

(v) Fizzling out

I never decided I was going to stop going, it just ended up fizzling out and never going back.

I missed one appointment and I got a letter saying I was discharged and I just never bothered to get referred back in.

Themes related to re‐engaging with dermatology care included

(i) Social media influence

I saw someone on Facebook showing pictures after a few weeks on the injection and I went straight to my GP for a referral back to the dermatologist.

I was on the Irish Skin Foundation page and I read about these new medicines and couldn’t believe there might be something that might work.

(ii) Novelty

After 60 years with eczema I just wanted to try something new, even if it killed me!

After going through the same creams and steroids and tablets in cycles for the last 40 years I thought that I had to at least try the new injection.

(iii) Exasperation with QOL

I just couldn’t keep going in the state I was in, every day was a struggle and I thought that even if the medicine worked a little bit it might make things bearable.

(iv) Life‐changing improvements seen with novel treatments.

My skin got better so quickly and I had to readjust to redirect my energy from scratching, it was really weird.

A few days after starting the tablets I left like Lazarus, I felt like I could finally start my life.

It was like standing up out of a wheelchair for the first time ever.

## 4 DISCUSSION

This study explores the reasons why adults with AD left dermatology services, explains why they re‐engaged, and deeply characterises the transformative effect that dupilumab therapy has had on QOL outcomes for patients with severe AD treated with dupilumab.

The decision of patients with severe AD to leave dermatology services because of ineffective treatments highlights the importance of having effective and well‐tolerated therapies for inflammatory dermatoses. Conversely, it shows that patient retention will be high if effective treatments are available and patients are satisfied with their care. As suggested in our previously studies examining BOT in adults and children with AD before the advent of dupilumab, effective systemic therapy dramatically reduced BOT, particularly related to needing to use less topical treatments.^4^, ^5^ Our previous work had identified that patients may consider infrequent injection therapy to be preferable to very frequent topical treatment,^4^, ^5^ which is consistent with the results of this study. Patients described genuinely life‐changing benefits after gaining control of their skin disease with dupilumab. There was no difference seen in those who had developed ocular or facial inflammation, suggesting that these side effects tend to be mild and considered tolerable. While the dermatology field has been blessed to have fantastically effective treatments for psoriasis for many years, with PASI 100 a potential treatment target even for severe disease,^18^ the therapeutic landscape for AD is only now beginning to catch up. This study also highlights that patients with severe AD had perceived that dermatologists had been frustrated by their inability to offer effective targeted treatments in the era before dupilumab. Therefore we hope that the advent of effective treatments will also enhance job satisfaction for dermatologists who look after patients with severe AD. Given the excellent safety profile of dupilumab, it is important to minimise the BOT associated with dupilumab prescription by avoiding unnecessary monitoring blood tests or excessively frequent monitoring visits. The expenses faced by patients and families should also be considered when prescribing novel therapeutics for AD, especially in health services where the prescription is not fully reimbursed.

Strengths of this study include the use of mixed methods for triangulation of data acquisition with quantitative data analysis informing qualitative data collection; the relatively severe cohort; the adequate sample sizes in both methods; the use of a questionnaire validated for broad application; and the use of semi‐structured interviews. Limitations of the study include the relatively low number of participants. However, dupilumab has only been funded relatively recently, so we captured a significant proportion of our patients who have been on dupilumab for a clinically meaningful duration. Pre‐dupilumab BOT reflections were retrospectively provided, which may have affected the statements provided. The BOT related to co‐morbidities such as food allergy, airways disease, and neuropsychiatric disease was not explored. This was a single centre study, so patients may have had a less diverse experience of different dermatology practices.

Dermatologists must continue to strive to enhance outcomes for our patients with severe AD. As adults with severe AD return to dermatology care on foot of the revolution in the landscape of therapeutics, the implications of increasing patient numbers needing biologic and JAKi therapy should be factored into healthcare and finance planning.

## CONFLICT OF INTEREST STATEMENT

The authors declare that they have no conflicts of interest.

## AUTHOR CONTRIBUTIONS

**Emma Porter**: Conceptualisation (equal); data curation (equal); formal analysis (equal); investigation (equal); methodology (equal); project administration (equal); resources (equal); software (equal); validation (equal); visualisation (equal); writing – original draft (equal); writing – review & editing (equal). **Cathal O’Connor**: Conceptualisation (equal); data curation (equal); formal analysis (equal); investigation (equal); methodology (equal); project administration (equal); resources (equal); software (equal); supervision (equal); validation (equal); visualisation (equal); writing – original draft (equal); writing – review & editing (equal). **Michelle Murphy**: Conceptualisation (equal); investigation (equal); methodology (equal); project administration (equal); resources (equal); software (equal); supervision (equal); validation (equal); writing – review & editing (equal)

## ETHICS STATEMENT

Ethical approval was granted by the Clinical Research Ethics committee of the Cork Teaching Hospitals.

## Data Availability

The data underlying this article will be shared on reasonable request to the corresponding author.
